# Influence of Nano-HA Coated Bone Collagen to Acrylic (Polymethylmethacrylate) Bone Cement on Mechanical Properties and Bioactivity

**DOI:** 10.1371/journal.pone.0129018

**Published:** 2015-06-03

**Authors:** Tao Li, Xisheng Weng, Yanyan Bian, Lei Zhou, Fuzhai Cui, Zhiye Qiu

**Affiliations:** 1 Department of Orthopedic Surgery, Nanfang Hospital, Southern Medical University, Guangzhou, 510515, PR China; 2 Department of Orthopedic Surgery, Peking Union Medical College Hospital, Peking Union Medical College, Beijing, 100730, PR China; 3 Department of Materials Science and Engineering, Tsinghua University, Beijing, 100087, PR China; University of Texas at San Antonio, UNITED STATES

## Abstract

**Objective:**

This research investigated the mechanical properties and bioactivity of polymethylmethacrylate (PMMA) bone cement after addition of the nano-hydroxyapatite(HA) coated bone collagen (mineralized collagen, MC).

**Materials & Methods:**

The MC in different proportions were added to the PMMA bone cement to detect the compressive strength, compression modulus, coagulation properties and biosafety. The MC-PMMA was embedded into rabbits and co-cultured with MG 63 cells to exam bone tissue compatibility and gene expression of osteogenesis.

**Results:**

15.0%(wt) impregnated MC-PMMA significantly lowered compressive modulus while little affected compressive strength and solidification. MC-PMMA bone cement was biologically safe and indicated excellent bone tissue compatibility. The bone-cement interface crosslinking was significantly higher in MC-PMMA than control after 6 months implantation in the femur of rabbits. The genes of osteogenesis exhibited significantly higher expression level in MC-PMMA.

**Conclusions:**

MC-PMMA presented perfect mechanical properties, good biosafety and excellent biocompatibility with bone tissues, which has profoundly clinical values.

## Introduction

Polymethylmethacrylate (PMMA) is an organic polymer material. Because of its good mechanical properties, operating performance and biological inertness[1–4], it is widely used in clinical applications such as vertebroplasty[5], arthroplasty[6, 7] and defect repair[8–10]. After implantation, aseptic loosening and shifting of a prosthesis is an important cause of surgical failure. Aseptic loosening of the prosthesis accounts for 52–55% of revision surgeries[11–13] and occurs most frequently at the cement-bone interface[14].

Several strategies have been employed to improve PMMA-based cement-bone interaction. These strategies have mainly focused on improving the material properties or the biological activity of the bone cement. Studies suggest that a lower elastic modulus can reduce failure rates while reducing stress shielding and graft shear stress[15]. The combined effect can eventually reduce bone tissue absorption, small movement and connective tissue formation at the interface, thereby reducing the risk of aseptic loosening[16–20]. A study by Litsky AS *et al* showed that the aseptic loosening rate significantly decreased after the elastic modulus of a PMMA bone cement was reduced from 2.1GPa to 0.27GPa[16].

Improving the biological activity of bone cement would enhance cement-bone crosslinking, thus reducing the loosening rate[21–24]. In addition, the load transferred at the cement-bone interface is mainly based on direct contact rather than on chemical bond formation. Improving the cement-bone crosslinking increases the contact area of the interface, thus making the load transfer more uniform. *In vivo* experiments by Miller MA *et al*. confirmed these theories. In the experiments, the interface from a retrieved human tibia prosthesis was analyzed; the results indicated that deeper crosslinking could greatly reduce the incidence of loosening[25].

The nano-HA coated bone collagen (mineralized collagen, patent number: ZL01129699.2, MC) is a combination material of recombinant human collagen and nano-hydroxyapatite (HA) that is made *in vitro*[26]. In previous studies, MC showed strong biological and osteogenic activities in bone defect repair[26, 27]. In addition, MC maintained its osteogenic activity after combination with calcium sulfate hemihydrate (CSH)[28–31] or polylactide (PLA)[31–34].

The present research investigates the mechanical properties and bioactivity of PMMA bone cement after the addition of nano-HA-coated bone collagen.

## Methods

### MC-PMMA bone cement preparation

All samples were prepared in accordance with International Standard (ISO5833[35]). Briefly, MC (Allgens Medical, Beijing) was added to commercial PMMA bone cement (Palacos MV, Heraeus) during the early period of dough phase, and then mixed thoroughly as MC-PMMA group. Commercial bone cement served as control (C-PMMA). After mixing, we set them into stainless steel molds with Φ6×12mm, Φ35×2mm, 5×5×5mm, 5×2×2mm to produce samples with different sizes. All samples were sterilized by 20kGy γ-ray irradiation.

### Mechanical properties (compressive strength, compression modulus and solidification)

Five bone cement specimens in each group were tested for compressive strength and compression modulus (Allround, Zwick/Roell, Germany) in accordance to ISO 5833[35].

Dough time and setting time were determined according to ISO 5833[35] and ASTM C191[36]. We divided the solidification phase into four periods including mixing, waiting, application and setting with commercial PMMA bone cement (Osteopal V and Mendec Spine) as controls.

### Biological properties

#### *In vitro* experiments

Preparation of material extracts: Samples of 5×5×5mm were submerged into normal saline (NS) and MEM-EBSS cell culture medium (containing 10% horse serum, 1% NEAA, 0.25Mm sodium pyruvate, provided by Cell Resource Center in PUMCH) respectively. After incubation at 37°C for 48h, the samples were adjusted to PH 7.4 and stocked in 4°C.

Cytotoxicity and proliferation test with CCK8 method: In the 96-well culture plates, 100μl MEM-EBSS with 5×10^3^ cells/ml of L929 mouse connective tissue cells (Cell Resource Center in PUMCH) was added except the reference group. 10μl MC-PMMA and C-PMMA extracts were added to experimental group and control group respectively, and 0.1% sterilized phenol was designed as positive control.

The cells were incubated in 37°C, 5% CO_2_ and cultured for 1, 3, 5 and 7 days. Then 10μl CcK8 reagent (Dojindo, Japan) was added in each well to measure the absorbance at 450nm (OD_450_) by the microplate reader (Thermo Multiskan Spectrum, Thermo Scientific, USA) after 2h incubation. The cell relative growth rate (RGR) was calculated by the formula below.

$$\text{RGR}=\;\left({\text{OD}}_{\text{test}}{\text{-OD}}_{\text{R}}\right)\;\text{/}\;\left({\text{OD}}_{\text{NC}}{\text{-OD}}_{\text{R}}\right)\;\times \;\text{100}\%$$

The cytotoxicity scale (CTS) was evaluated according to ISO 10993 [37].

Hemolysis test: Fresh diluted rabbit blood (8ml fresh rabbit blood and 10ml saline) was used to investigate hemolysis of material extracts. 10mL MC-PMMA and C-PMMA extacts were added to 0.2mL fresh diluted rabbit blood respectively with distilled water as positive control and NS as negative control. After incubated at 37°C for 60min and centrifuged for 5min (2500r/min), the absorbance at 545nm (OD_545_) for the supernatant was measured to calculate the hemolysis ratio (HR).

$$\text{Hemolysis}\;\text{Ratio}\;=\;\left({\text{OD}}_{\text{test}}-{\;\text{OD}}_{\text{NC}}\right)\;/\;\left({\text{OD}}_{\text{PC}}-{\;\text{OD}}_{\text{NC}}\right)\;\times \;100\%$$

Osteogenesis test: Real-time polymerase chain reaction (RT-PCR) was performed to detect gene expression of osteocalcin (BGLAP), osteonectin (SPARC), collagen -1 (Col1A1) and bone sialoprotein (IBSP). For this experiment, bone cement samples with the size of Φ35×2mm were co-cultured with human osteosarcoma cells (MG 63) in the 6-well plate. After incubation at 37°C, 5% CO_2_ for 1, 7 and 14 days, the cells were collected using trypsin EDTA (provided by Cell Resource Center in PUMCH) and washed by PBS buffer.

Then the total RNAs from the collected cells were isolated by Trizol Reagent (Invitrogen Life Technologies, USA). The cDNA from the isolated RNA was reversely transcribed by RevertAid First Strand cDNA Synthesis Kit (Thermo, USA) and subjected to RT-PCR by using FastStart Universal SYBR Green Master (Rox) (Rothe, Switzerland). RT-PCR subjected the samples to the following condition: pre-denaturation 95°C/10 min, run through 40 cycles (denaturation 95°C/15s, annealing and extension 60°C/60s), terminal extension 60°C/5min and then melting analysis from 75°C to 95°C at ramp rate of 1°C /20s. The reference gene was ACTIN. The primers for gene SPARC, IBSP, Col1A1, BGLAP and ACTIN were designed with Primer Premier 5.0 (Canada) and synthesized (Invitrogen Biotechnology Co., LTD).

#### *In vivo* experiments

Prior to the experiments, approval was acquired by the Committee on the Use of Live Animals in Teaching and Research of the Peking Union Medical College Hospital. Licence to conduct experiments was issued by Peking University Laboratory Animal Centre. All the animals were operated and sacrificed under general anesthesia with injection of pentobarbital sodium(Sigma, US).

Acute systemic toxicity test (intraperitoneal injection): 30 Kunming mice weighing between 27–32g were divided randomly into two groups. The mice in experimental group and control group were injected respectively with MC-PMMA extracts and saline at 50ml/kg of body weight by intraperitoneal. Then we observed the general condition of mice (including respiration, body temperature, appetite and movement), weight change and toxic manifestations (such as vomiting, diarrhea, convulsions *et al*.) before administration and 24h, 48h, 72h after administration. The mice were sacrificed in 72 hours by inhaling of excessive carbon dioxide, and their livers and kidneys were further under histological observation. The acute systemic toxicity was evaluated according to ISO 10993[37].

Local reactions and chronic liver and kidney toxicity after implantation: 30 6-week-old SD rats with fasting food and water for 12h before operation were anaesthetized by intraperitoneal injection of 2% pentobarbital sodium at 20mg/kg of body weight. Then a 2cm sagittal incision was made on the scalp from 2cm above the posterior superior iliac spine, the epimysium and muscle fibers were separated along the direction of the gluteus maximus to form a cavity. The 5 × 2 × 2mm MC-PMMA bone cements were implanted in bilateral sizes and the incisions were closed in layers. After 1w, 4w, 7w, 12w and 26w of implantation, the muscle samples were drawn from six rats sacrificed by an intraperitoneal overdose injection of pentobarbital sodium, and under fixation by 4% paraformaldehyde, dehydration, embedment in paraffin, section and HE staining. Then the inflammatory condition and tissue reaction surrounding materials were examined with the light microscope. After 12 weeks of operation, liver and kidney were removed from sacrificed rats and observed in gross and biopsy.

Implantation in rabbit bone tissue: 18 adult New Zealand rabbits weighing 2–3kg were divided randomly into experimental group and control group. After fasting food and water for 12h, the rabbits were anaesthetized by ear vein injection of 3% pentobarbital sodium at 30mg/kg of body weight. Then we fixed the rabbits, cut the hair of the legs, disinfected the skin with chlorhexidine and performed the operation under sterile condition. Then a 2cm sagittal incision was made on the skin of femoral distal end the muscles were separated bluntly to expose medial and lateral femoral condyle. A cylindrical cavity (3mm in diameter and 5mm in depth) was made on the medial femoral condyle by using bone drill and irrigated with saline continuously during the drilling to avoid local high temperature. The rabbits in experimental group were implanted with MC-PMMA bone cements, while those in control group implanted with C-PMMA bone cements. Finally the wounds were sutured with vicryl and conventional feeding was performed after implication.

Micro-CT: The material-embedded rabbit bone tissues were harvested after sacrificed with 100 mg/kg pentobarbital sodium at 4th, 12th and 24th week respectively and imaged with three-dimensional microfocus computed tomography (micro-CT, Siemens Inveon MM Gantry CT, Germany), at a voltage of 60 kV and an electric current of 100μA. The fusion situations between embedded materials and surrounding bone tissues were observed in the images of cross-sectional slices of the samples.

Histological Analysis: 3 New Zealand rabbits from experimental group and control group were sacrificed with 100 mg/kg pentobarbital sodium after 4, 12 and 24 weeks respectively. The material-embedded rabbit bone tissues were harvested, fixed in 4% paraformaldehyde, dehydrated and embedded with resin. A total of 50μm sections were obtained with hard tissue slicer (Leica Cryocut, Germany). And the sections were stained with Van Gieson’s staining. The sections were observed under light microscope to assess the bone-cement interface crosslinking situation. The area of bone tissue growing into bone cement was measured by observation of three sections chosen for each sample with Image-Pro Plus 6.0 (Media Cybernetics, USA), then its proportion of the whole bone cement could be calculated. The Affinity Index of bone (AI)[38], which was the proportion of length between bone tissue and bone cement to the total length of the interface, was also measured using Image-Pro Plus 6.0.

### Statistical analysis

Statistical analysis was performed with SPSS17.0. Differences between groups were tested by a one-factor analysis of variance (ANOVA) and differences between two means were examined by *t* test. P<0.05 was considered statistically significant.

## Results

### Evaluation of mechanical properties

#### Compressive strength and compression modulus

The compressive strength and compression modulus of MC-PMMA in different proportions were shown in Table 1. We found that the compressive strength decreased and the compressive modulus declinedwith increasing MC content. 15.0% (wt) impregnated MC-PMMA indicated minimal compressive strength of 89.30±5.26MPa and compression modulus of 1.21±0.12GPa, which was higher than the industry standard (no less than 70MPa according to ISO 5833[35] and ASTM F451[39]). 15.0% (wt) impregnated MC-PMMA had the best material properties and was used in the further experiments.

**Table 1:** The compressive strength and compressive modulus of MC-PMMA in different proportions ($\overset{-}{X}$± SD, n = 5).

MC proportion (wt/wt%)	compressive strength(MPa)	compression modulus (GPa)
0	90.53±4.39	1.91±0.08
5.0	90.10±2.90	1.84±0.09
10.0	90.16±2.00	1.52±0.09
15.0	89.30±5.26	1.21±0.12
20.0	88.03±4.74	1.63±0.07

#### Solidification

**Fig 1:**
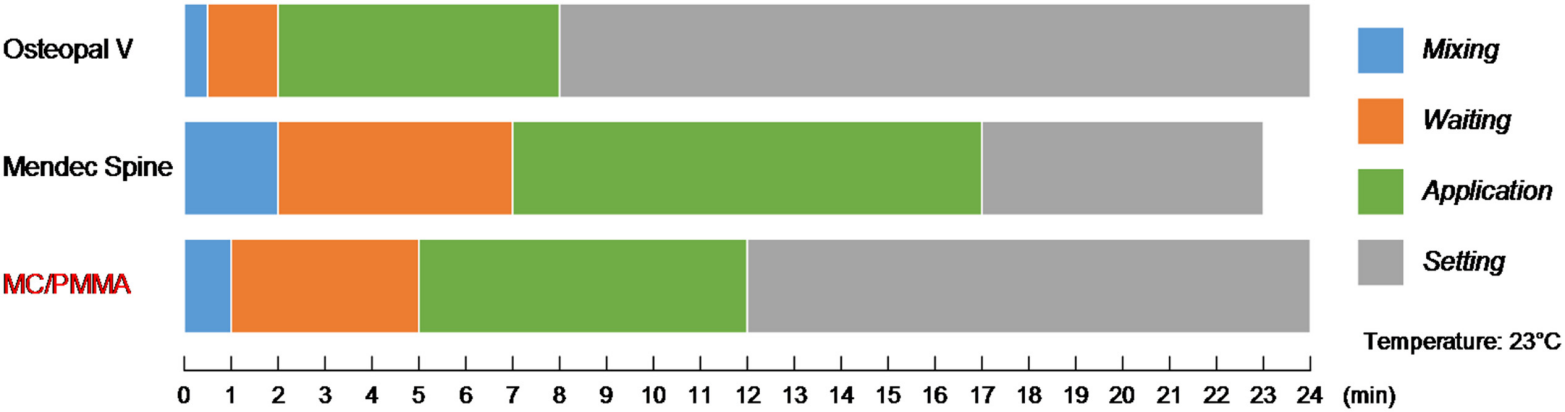
Solidification characteristics of MC-PMMA. Measured at 23°C with PMMA bone cement (Osteopal V, Mendec Sphe) without MC as control.

The solidification characteristics of MC-PMMA at 23°C were displayed in Fig 1. The mixing period, waiting period, application period and setting period were 1min, 4min, 7min and 12 min respectively with PMMA bone cement (Osteopal V and Mendec Spine) as controls, which suggested that MC-PMMA had no influences on its operation performance.

### Evaluation of biosafety

#### Cytotoxicity and cell proliferation

The growth states in 24h and 5 days after L929 cell subculture were observed. We discovered the cells in all groups except the positive control group were in good condition without cell necrosis debris.

The cell relative growth rate (RGR) with CKK8 method in 1day, 3 days, 5 days and 7 days after L929 cell subculture was calculated. The RGR of MC-PMMA was above 90% in 1st week and with 0–1 cytotoxicity scale, which implied the MC-PMMA had no toxic effect on cells.

#### Blood compatibility test

The 6 tubes in positive control group were red uniformly and no red blood cells were residual in the bottom of the tubes, which illustrated that no hemolysis occurred. All red blood cells in the negative control tubes deposited and the supernatant was clear, which suggested no hemolysis. We observed the similar phenomenon in both MC-PMMA group and C-PMMA group.

The absorbance at 545nm wavelength (OD_545_) and hemolysis ratio (HR) for different groups were calculated. The HR of MC-PMMA and C-PMMA were 0.26 ± 0.08% and 0.18 ± 0.11% respectively, both of which were less than 5%. Based on the results, it suggested that MC-PMMA and C-PMMA couldn’t cause hemolysis.

#### Short-term acute systemic toxicity

There was no movement decrease, ptosis, difficulty breathing, diarrhea, cyanosis, tremors or any other toxic symptoms happened in the mice before or after intraperitoneal injection of material extracts. The weight change was insignificant (P> 0.05). HE staining of liver and kidney at 72h showed normal cell morphology without degeneration or necrosis (S2 Fig).

#### Local reactions and chronic liver and kidney toxicity after implantation

After implantation for 1, 4, 7, 12 and 26 weeks, the incision healed without infection or abscess. HE staining of the local muscle showed inflammatory cells infiltration at 1 week, and then gradually reduced with fibrous connective tissue formation. A dense fiber wrapped around the material after 12 weeks (S3 Fig). At 12 weeks, HE staining of liver and kidney showed normal cell morphology without degeneration or necrosis (S4 Fig).

#### Evaluation of bone tissue compatibility

**Fig 2:**
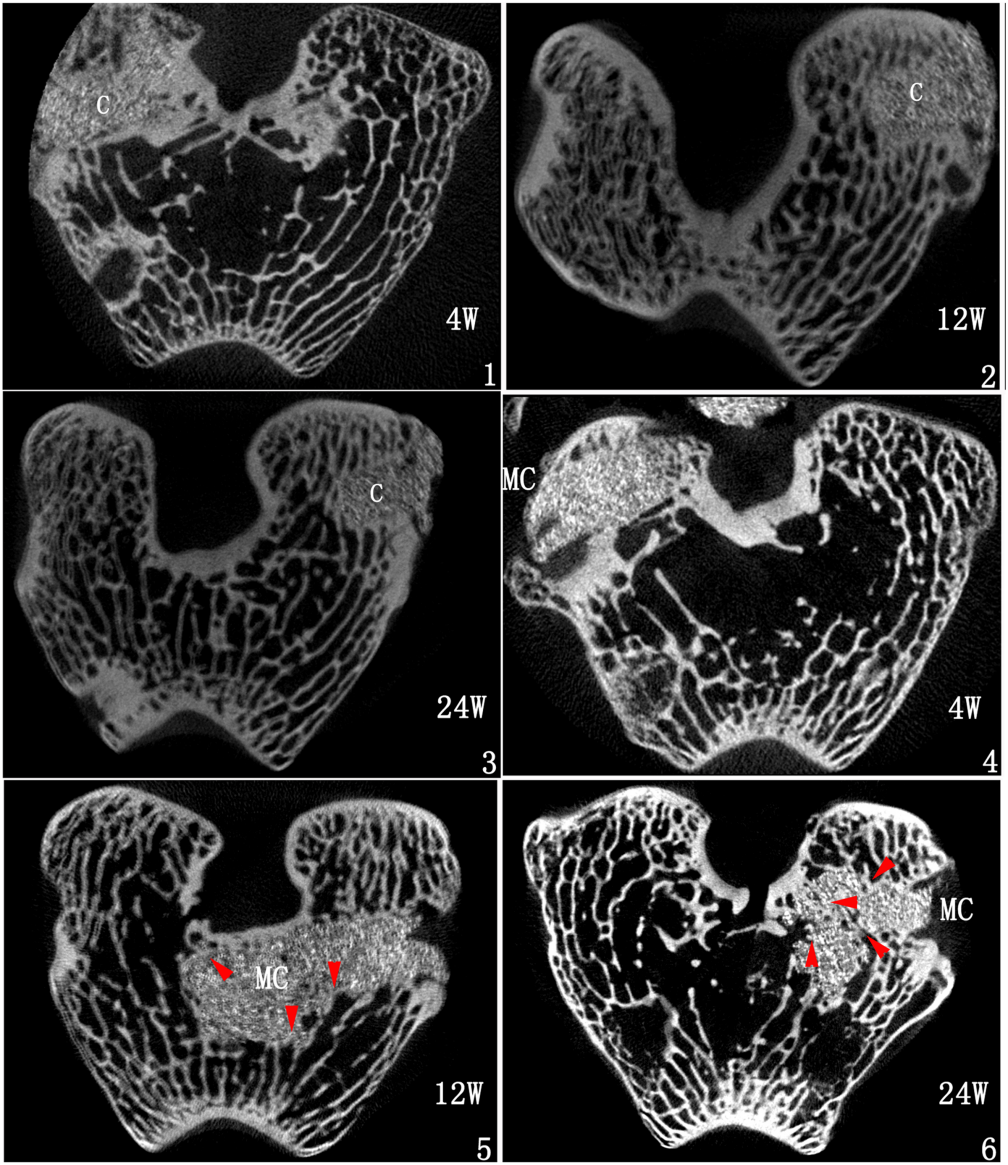
Micro-CT of C-PMMA (1–3) and MC-PMMA (4–6) after implantation for 4 (1,4), 12 (2, 5) and 24 weeks (3, 6). Red arrow indicates the insertion site of trabecular; C, C-PMMA; MC, MC-PMMA.

Micro-CT evaluation: Micro-CT evaluation of the samples after implantation for 4, 12 and 24 weeks showed almost no shedding of cement or tiny fracture (Fig 2). The interface was sharp and clear in the C-PMMA group, almost no trabecular ingrowth into the cement. In MC-PMMA group, the surface was similar to that of C-PMMA at 4 weeks, but more and more bone tissue grew into cement from 12 to 24 weeks, forming a better interface interspersed integration.

**Fig 3:**
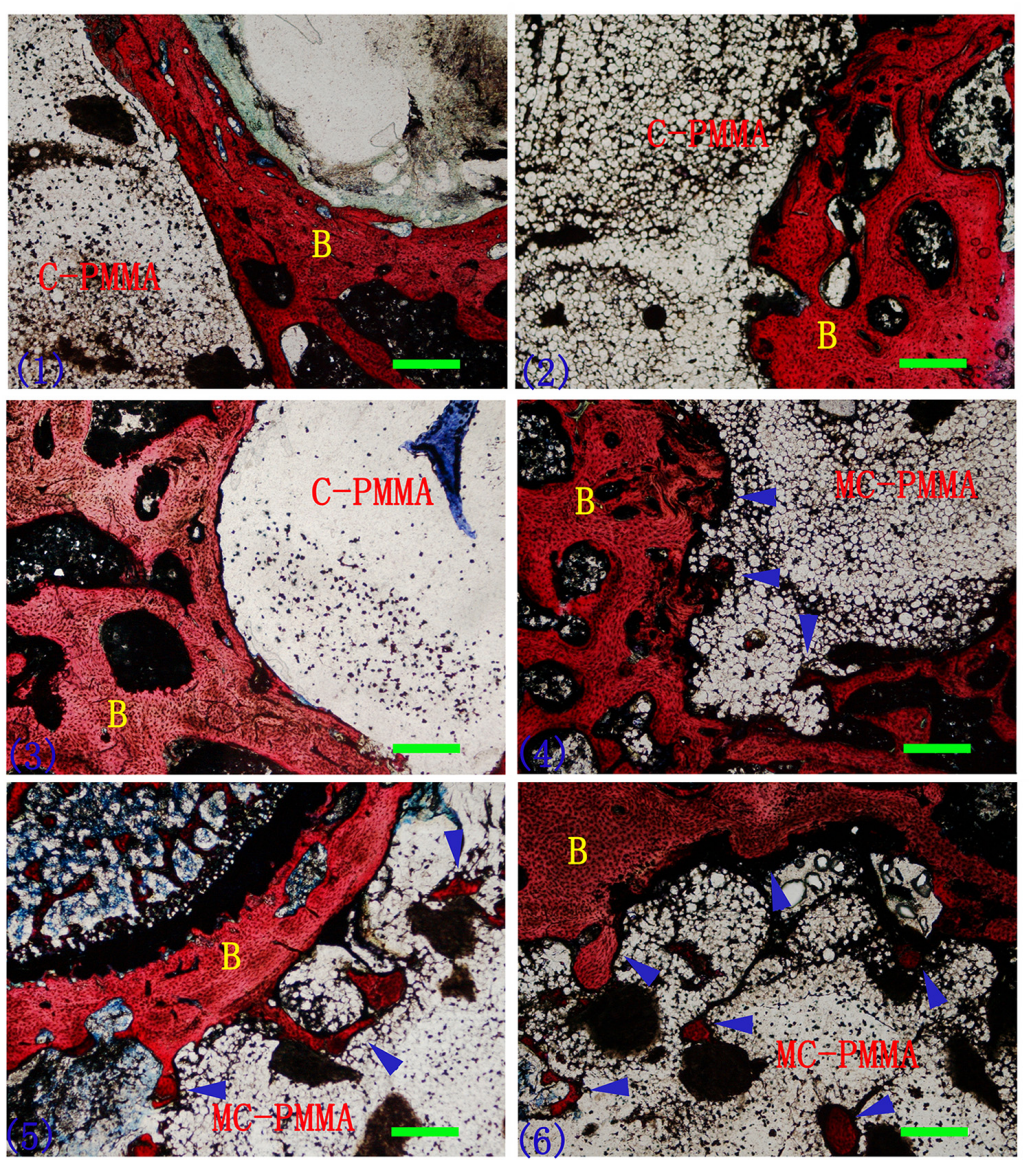
Van Gieson surface staining of C-PMMA (1–3) and MC-PMMA (4–6) after implantation for 4 (1,4), 12 (2, 5) and 24 weeks (3, 6). Blue arrow indicates the insertion site of bone tissue; B, bone; Bar = 400μm.

Histological evaluation: Van Gieson surface staining showed that there was almost no shedding of cement or tiny fracture in any of the sample (Fig 3). There was more bone tissue ingrowth into cement forming a firm anchoring in MC-PMMA and bone insertion depth increased with prolonged implantation.

**Fig 4:**
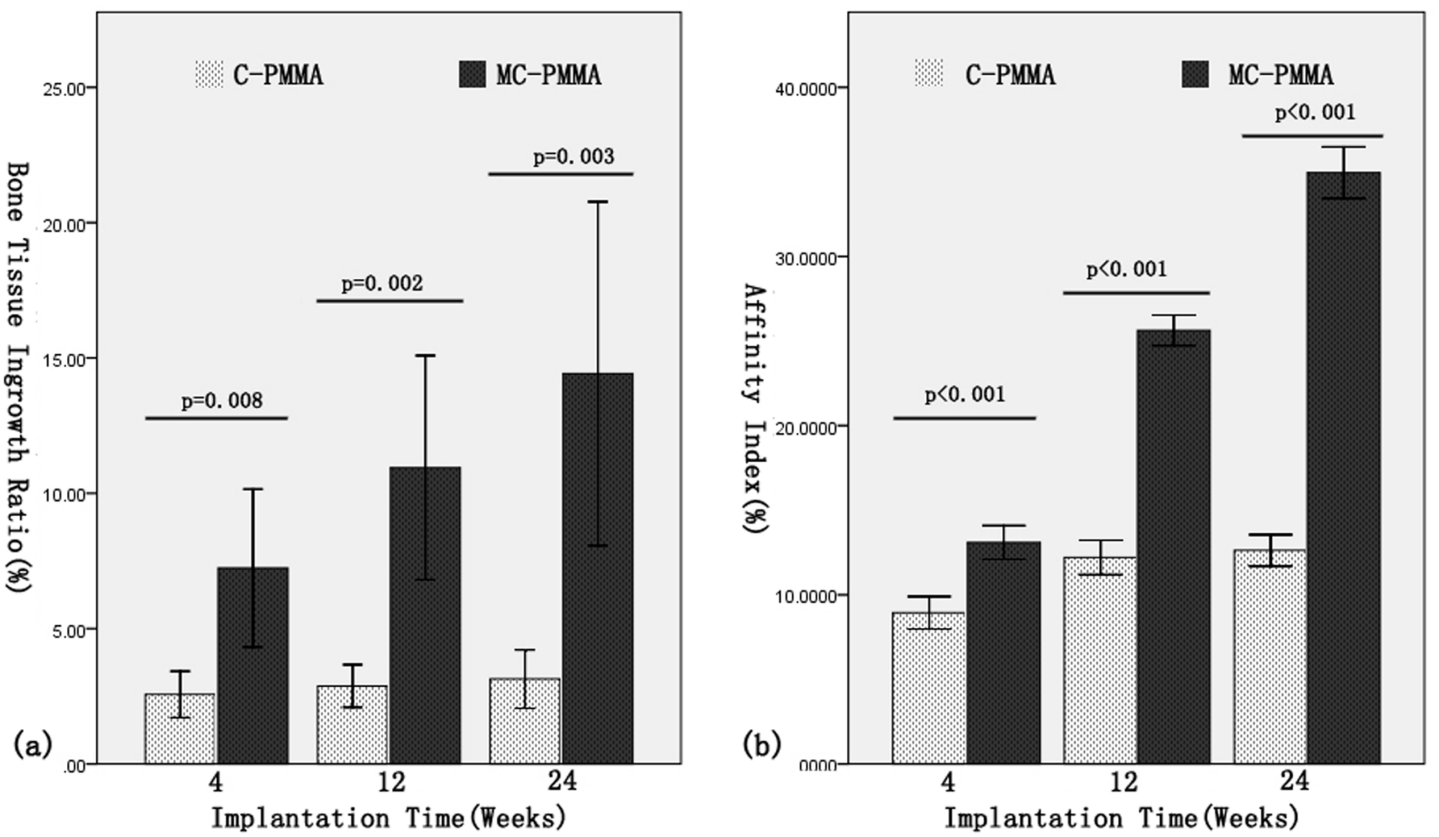
Bone tissue ingrowth ratio (A) and affinity index (B) for all the tested cements for 4, 12 and 24 weeks. $\overset{-}{X}$±SD, n = 9. (A) The bone tissue ingrowth ratios in MC-PMMA were significantly higher than that of C-PMMA group (p<0.05). (B) The bone affinity indexes (AI) in MC-PMMA were significantly higher than that of C-PMMA group (p<0.05).

The bone tissue ingrowth ratios in MC-PMMA group were 7.24±4.46%, 10.95±6.34% and 14.42±9.72% after implantation for 4, 12 and 24 weeks respectively, which were higher than that of C-PMMA group (2.57±1.31%, 2.88±1.20% and 3.14±1.65%, respectively, p<0.05) (Fig 4A). The bone affinity indexes (AI) in MC-PMMA group were 13.10±1.52%, 25.63±1.38% and 34.96±2.33% respectively, significantly higher than the corresponding C-PMMA group (8.94±1.47%, 12.21±1.56% and 12.62±1.42%, respectively, p<0.05) (Fig 4B).

**Fig 5:**
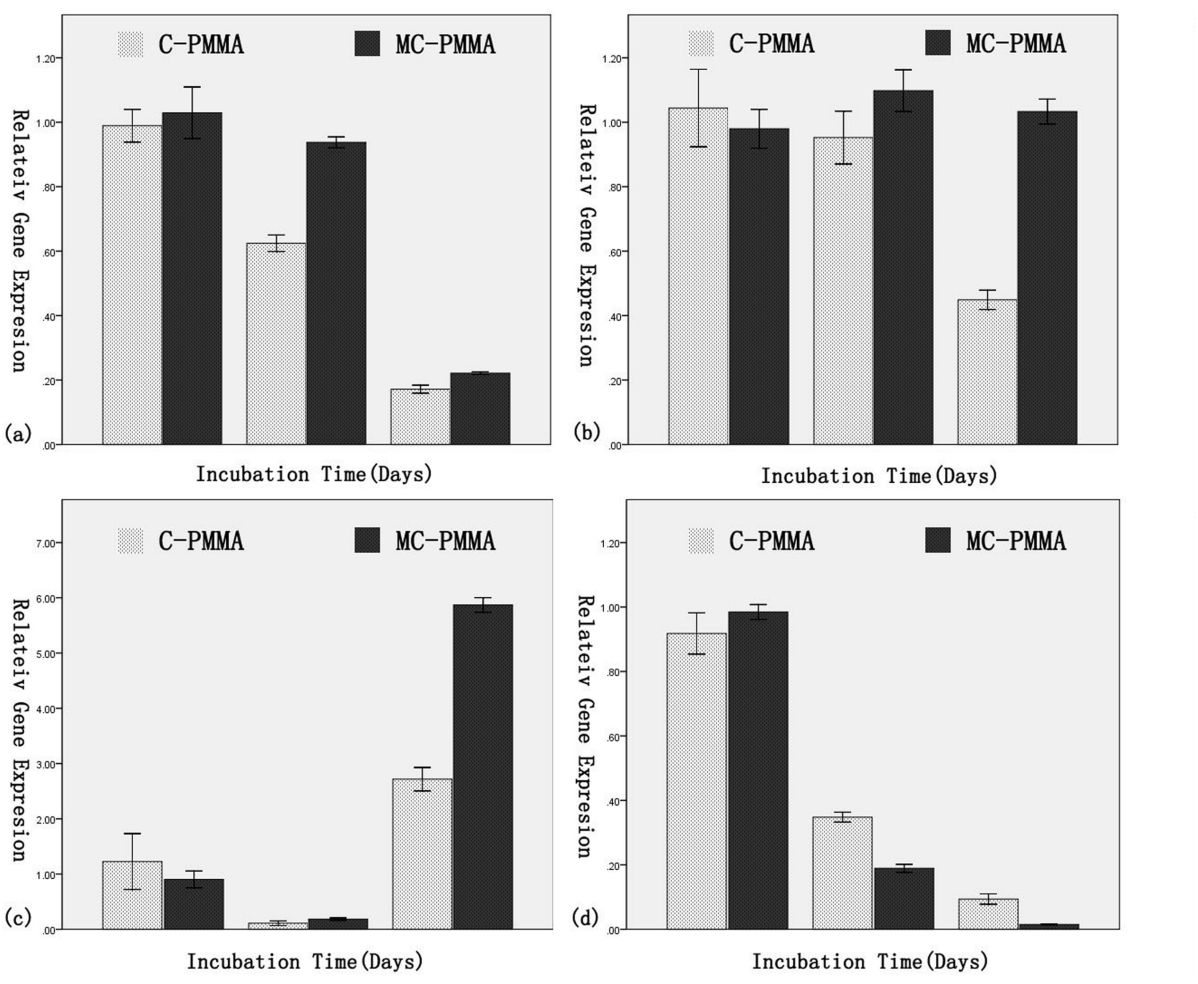
Real-time PCR analysis of osteogenic gene markers expression of MG63 cell line grown in the surface of C-PMMA and MC-PMMA after 1, 7 and 14 days. (a) Osteonectin (SPARC) (b) Collagen-1 (Col1A1) (c) Bone sialoprotein (IBSP) and (d) Osteocalcin (BGLAP). Expression normalized to housekeeping gene ACTIN. X±SD, n = 3, p<0.05.

Osteogenesis evaluation: The synthesized cDNA from the extracted RNA of the MG63 cells grown in the surface of the samples was used for detecting genes expression. The resulting levels of gene expression were reported as fold change (2^-ΔΔCt^) in relation to the expression levels recorded from control calibrator group. The result in Fig 5 shows the cellular gene expression of the cells collected from C-PMMA and MC-PMMA samples. After 1 day of incubation, relatively insignificant difference was observed between the two samples across the four primers. The cells from MC-PMMA had depressed expression of BGLAP and higher expressions of SPARC, Col1A1 and IBSP than the cells from C-PMMA after further incubation for 7 and 14 days. Significant differences in the amount of gene expression were observed between C-PMMA and MC-PMMA, especially in the expression of Col1A1 and IBSP after 14 days, wherein MC-PMMA registered a value of (1.03±0.03 and 5.87±0.12 respectively), which was almost double of that of C-PMMA (0.45±0.03 and 2.72±0.90 respectively).

## Discussion

### Mechanical properties

Compressive strength, elastic modulus and performance are the most important properties of bone cement. PMMA has outstanding advantages with respect to its compressive strength and operating performance; however, it has the disadvantage of a high elastic modulus, which results in stress shielding and implant loosening[40, 41]. MC-PMMA exhibited excellent operatingperformance and compressive strength. In addition, the elastic modulus of MC-PMMA (15%, wt/wt) was reduced from 1.91±0.08GPa to 1.21±0.12GPa, which was closer to that of cancellous bone (0.05–0.5GPa)[42].

Compared with various bioactive substitute materials such as HA and AWG, MC-PMMA had a greater advantage in its elastic modulus (Table 2). In addition, many PMMA-based bioactive cements have been developed. In contrast, the Ti-PMMA developed by Fukuda C *et al* had sufficient compressive strength but a high elastic modulus[43]. The HEMA-PMMA developed by Wolf-Brandstetter C, The HA-chitin-PMMA developed by Kim SB and the TCP-PMMA developed by Vazquez B all showed reductions both in compressive strength and elastic modulus[44–46].

**Table 2:** Mechanical properties of different materials.

Author	Material	Strength(MPa)	Elastic modulus(GPa)
Ascenzi A[71, 72]	Osteon	102–120	5.5–12
Rho JY[73]	Trabecular	-	1–20
Kokubo T[42]	Cancellous bone	2–12	0.05–0.5
	Cortical bone	100–230	7–30
	Bioglass(45S5)	-	35
	HA	500–1000	80–110
	Apatite-and wollastonite-containing glass (AWG)	1080	118
Fukuda C[43]	Ti-PMMA	126.5–130.0	3.45–4.19
Wolf-Brandstetter C[44]	HEMA-PMMA	75–80	1.3–1.4
Kim SB[45]	HA- chitin-PMMA	48.8–81.8	1.5–2.4
Vazquez B[46]	TCP-PMMA	76–80	1.4–1.6
Our Study	MC-PMMA	89.30±5.26	1.21±0.12
	PMMA	90.53±4.39	1.91±0.08

### Biosafety

Biosafety is a prerequisite for bone substitute materials *in vivo* applications. In accordance with the international standards ISO 10993[37], the MC-PMMA bone cement was biologically safe in cytotoxicity and proliferation assays and with respect to blood compatibility, local reaction after implantation, acute systemic toxicity and chronic liver and kidney toxicity.

Previous studies of MC have shown no toxic effects[26]. The toxicity of bone cement is mainly due to the toxicity of its components and of unpolymerized monomer. Adding MC to the PMMA component did not increase the toxicity, indicating that there were no significant effects of MC on PMMA polymerization.

Because bone cement will remain in the body for long periods, the long-term toxicity of MC-PMMA applications requires further evaluation.

### Biocompatibility with bone tissue

#### Interface morphology

Charnley and Willert studied the interfacial histological reaction in the 1970s and discovered a pearl-like layer between bone cement and bone[1, 47]. Draenert and Linder then revealed that this layer was demineralized bone tissue with dense connective tissue in the inner layer[2, 48]. These findings are consistent with the histological features observed in our study (Fig 3). Both C-PMMA and MC-PMMA showed connective tissues between the bone structure and the bone cement.

The formation of connective tissue is related to cement shrinkage that occurrs during the polymerization reaction. A study by Muller *et al*. found a volume change of 2–6% during shrinkage[49], which produced voids in the interface. The existence of voids caused slight movement and absorption in the interface and ultimately induced the formation of a layer of dense connective tissue. It was difficult to avoid the formation of connective tissue.

#### Interface osteonecrosis

Bone tissue necrosis usually occurs after implantation of PMMA bone cement. The necrosis is caused mainly by the exothermic polymerization reaction and by the toxicity of MMA monomers[3].

In our study, both C-PMMA and MC-PMMA caused apparent tissue necrosis. This result indicated no significant effect of MC on PMMA polymerization or the exothermic reaction. However, the extent of the exothermic reaction was volume-related. Stanczyk *et al* revealed that the interface would produce a high temperature above 70°C only when the implanted volume exceeded 10% of the bone tissue volume[50]. Boner *et al*. found that the thermal necrosis risk increased only when the implanted PMMA thickness exceeded 5mm and the volume exceeded 6cc[51]. In our study, the implanted bone cement was less than 1cc in volume and less than 5mm in thickness.

#### Interface crosslinking

Interface crosslinking has been the greatest concern in materials research. In our study, we observed more bone tissue ingrowth into the bone cement to form tight crosslinking, and the bone ingrowth ratio gradually increased with the duration of implant residence. After 24 weeks, the bone ingrowth ratio in the MC-PMMA group was four times that in the C-PMMA group (Fig 4).

Many studies of PMMA-based cements have attempted to enhance interface crosslinking. Current research ideas mainly include three strategies: (a) adding partially biodegradable materials, such as polyhydroxyalkenonate (PHA) and poly-β-hydroxybutyrate (PHB)[52, 53]; (b) adding completely biodegradable materials to commercial PMMA or modified PMMA, such as sodium fluoride[54, 55], nanoscale aluminum oxide (average size of 70nm)[56], cellulose[57, 58] and tricalcium phosphate ceramic (TCP)[59]; and (c) adding bioactive materials to commercial PMMA or modified PMMA, such as apatite wollastonite glass (AWG)[60, 61], hydroxyapatite (HA) [62, 63] and recombinant growth factor [64]. In all of these studies, the formation of pores and the induction and adsorption properties were responsible for improvements in interface crosslinking.

We compared the bone affinity index 12 weeks after implantation with representative PMMA-based studies. He Q *et al*. investigated porous PMMA bone cement created by adding cellulose, which resulted in a bone affinity index of 13.95%[65]. Goto K *et al*. investigated PMMA with the addition of titanium, indicating a bone affinity index of 11.0%[66]. Wong CT *et al*. investigated strontium and HA composited PMMA, which showed a bone affinity index that increased to 73.55%[22].

Because of the poor biological activity of PMMA, there have also been a large number of studies investigating non-PMMA-based bone cement. Kaili *et al*. studied the implantation of calcium silicate bioactive bone cement with the addition of strontium, which showed a 4.4% increase in the bone mass after 4 weeks[67]. Ulrich *et al*. investigated calcium phosphate bone cement with the addition of strontium, which showed a 6.8% increase of the bone mineralization proportion after 6 weeks of implantation[68].

MC-PMMA showed better biological activity than any of the above studies, with the exception of Sr-HA-PMMA. It was reasonable for MC to be biodegradable[26, 32], thereby forming a porous surface and thus providing a bone tissue growth scaffold. On the other hand, gene expression analysis showed increases in IBSP, SPARC and Col1A1 and decreases in BGLAP in the MC-PMMA group (Fig 5), indicating osteoinductive activity.

IBSP, SPARC, Col1A1 and BGLAP genes are expressed during proliferation and mineralization in osteocytes. Owen TA and Jääskeläinen T revealed that BGLAP expression decreased only during proliferation by Fos-Jun inhibition[69, 70]. It is thus reasonable to speculate that the impact of MC-PMMA on osteogenic activity is mainly to promote proliferation.

The pore formation and osteogenic activity of MC-PMMA might be involved in cement-bone interface integration; however, further studies are needed to investigate MC-PMMA porosity before and after implantation.

## Conclusions

PMMA bone cement with the addition of nano-HA-coated bone collagen showed superior mechanical properties, good biosafety and excellent biocompatibility with bone tissues. MC-PMMA (15.0%(wt) impregnated) significantly lowered the compressive modulus of PMMA from 1.91GPa to 1.21GPa while having little effect on its compressive strength and solidification, which could reduce the risk of aseptic loosening. In accordance with the international standard ISO 10993, MC-PMMA bone cement was biologically safe in cytotoxicity and proliferation assays and with respect to blood compatibility, local reaction after implantation, acute systemic toxicity and chronic liver and kidney toxicity. In addition, the bone-cement interface crosslinking was significantly higher for MC-PMMA than for C-PMMA 6 months after implantation in the distal femur of rabbits. A comparison of gene expression between the MC-PMMA and C-PMMA groups also suggested that MC-PMMA might improve osteogenic bioactivity. We propose that PMMA bone cement withadded nano-HA-coated bone collagen has profound clinical values.

## Supporting Information

S1 Fig The growth state in 24h and 5 days after L929 cell subculture. 1–4 was the growth state in 24h and 5–8 was for 5 days. The arrow indicated dead cells and no cell necrosis occurred in NC, MC-PMMA, C-PMMA groups. Phase contrast microscope (Olympus, Japan), 20×. NC: Negative Control; PC: Positive Control; MC-PMMA: Mineralized Collagen PMMA Bone Cement; C-PMMA: Classical PMMA Bone Cement.
(TIF)

S2 Fig HE staining of mice liver (1,2) and kidney (3, cortex; 4, medulla) after intraperitoneal injection of material extracts for 72 hours. 1, 10×; 2–4, 20×.
(TIF)

S3 Fig HE staining of local muscle after implantation of MC-PMMA for 1 (1), 4 (2), 7 (3), 12 (4) and 26 (5) weeks. Bar = 100μm; M, muscle; white arrow, inflammatory cells; black arrow, connective tissue.
(TIF)

S4 Fig HE staining of rat liver (1,2) and kidney (3, cortex; 4, medulla) after implantation of MC-PMMA in the gluteus maximus for 12 weeks. 1, 10×; 2–4, 20×.
(TIF)

S1 Table Information of gene primers. (DOCX)

S2 TableThe cell relative growth rate (RGR) with CKK8 method in different periods after cell subculture ($\overset{-}{X}$± SD (CTS), n = 6, %).(DOCX)

S3 TableThe absorbance at 545nm wavelength (OD545) and hemolysis ratio ($\overset{-}{X}$± SD, n = 6).(DOCX)

S4 TableWeight change after intraperitoneal injection of material extracts (gram, $\overset{-}{X}$±SD, n = 15).(DOCX)
