# Supplementary figures and images for: Influence of Nano-HA Coated Bone Collagen to Acrylic (Polymethylmethacrylate) Bone Cement on Mechanical Properties and Bioactivity
Source: PLoS One. 2015 Jun 3;10(6):e0129018. doi: 10.1371/journal.pone.0129018 (PMC4454564; doi:10.1371/journal.pone.0129018)

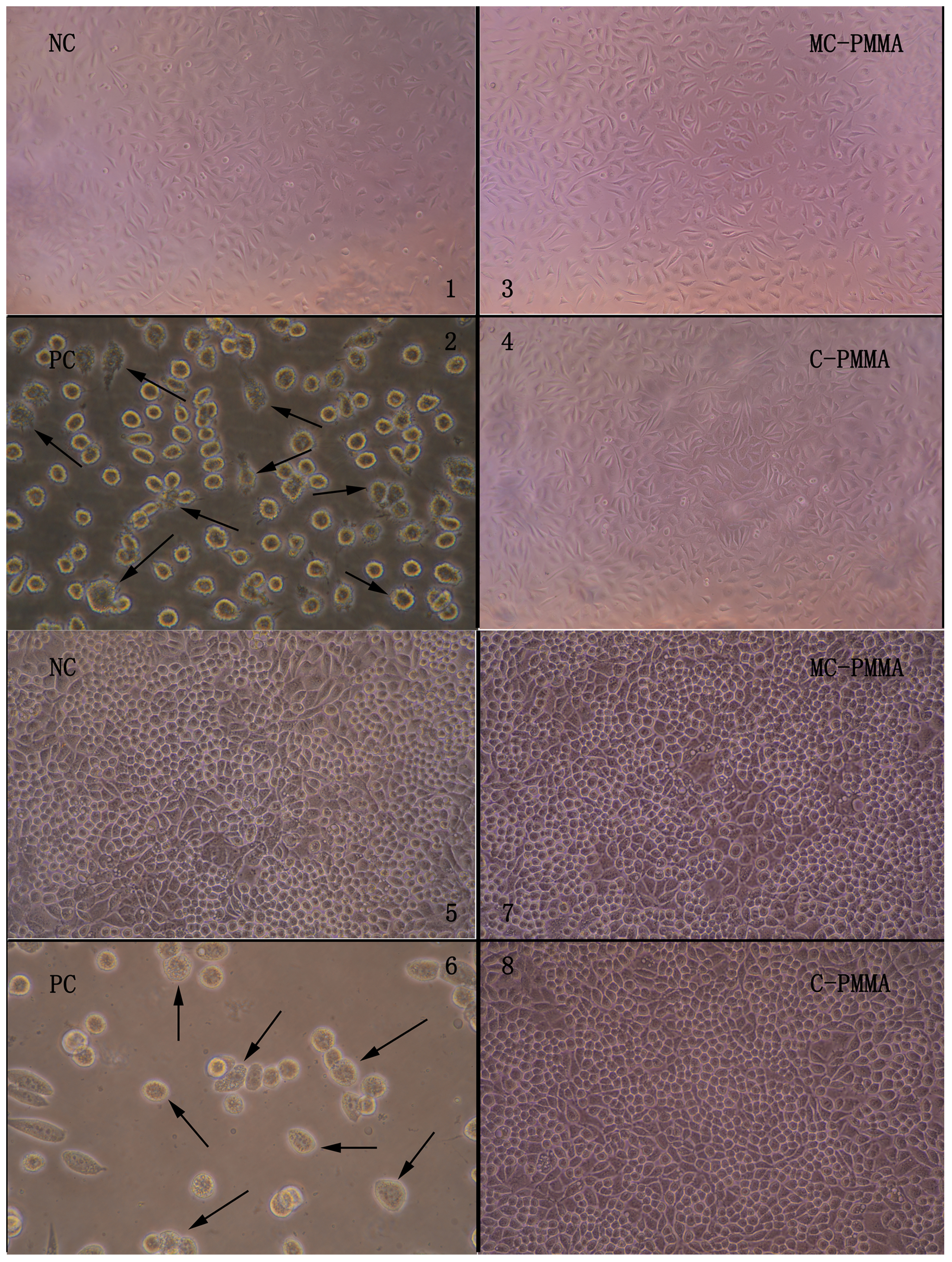

Supplement: S1 Fig — 1–4 was the growth state in 24h and 5–8 was for 5 days. The arrow indicated dead cells and no cell necrosis occurred in NC, MC-PMMA, C-PMMA groups. Phase contrast microscope (Olympus, Japan), 20×. NC: Negative Control; PC: Positive Control; MC-PMMA: Mineralized Collagen PMMA Bone Cement; C-PMMA: Classical PMMA Bone Cement. (TIF) [file pone.0129018.s001.tif]

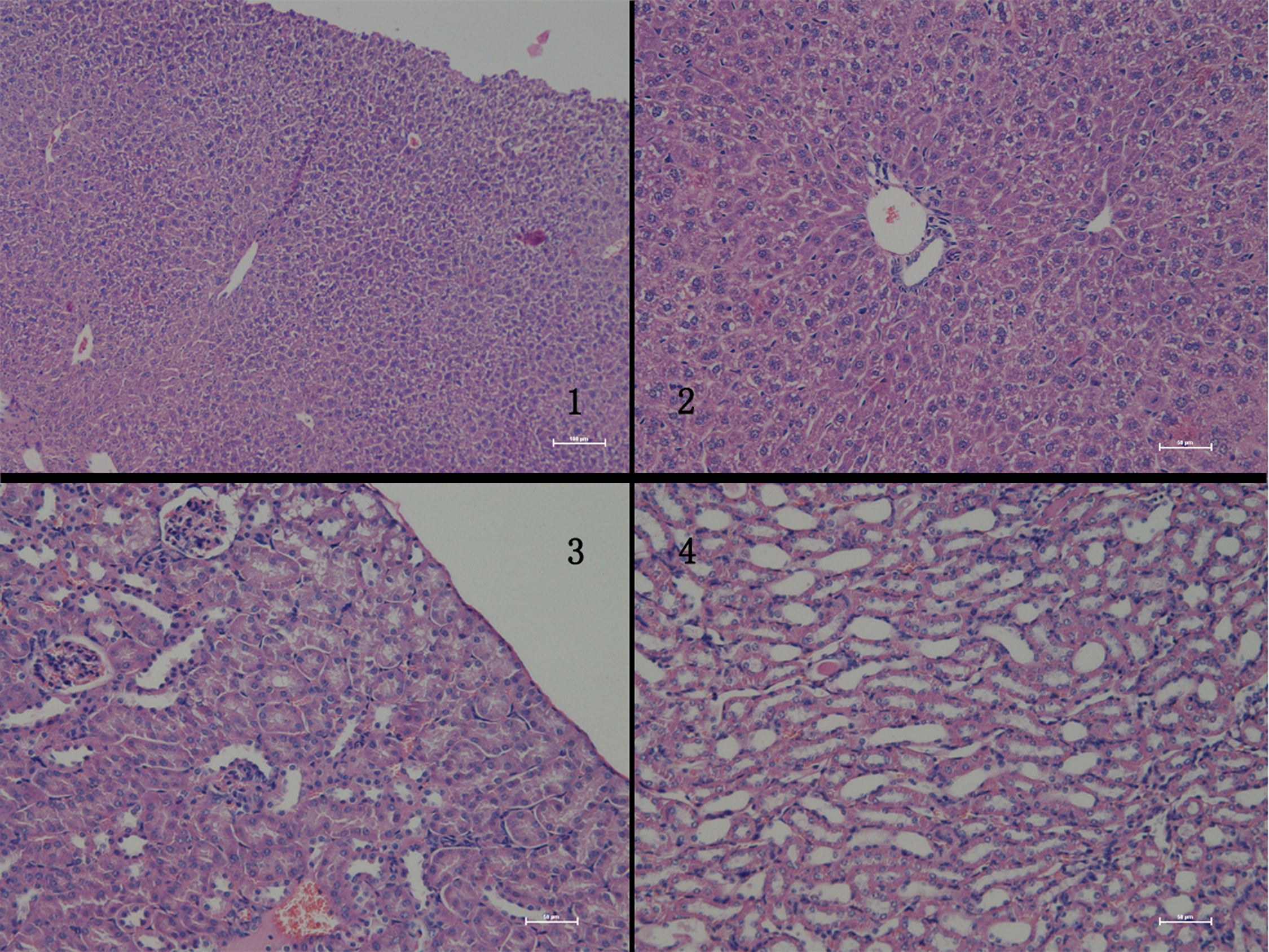

Supplement: S2 Fig — 1, 10×; 2–4, 20×. (TIF) [file pone.0129018.s002.tif]

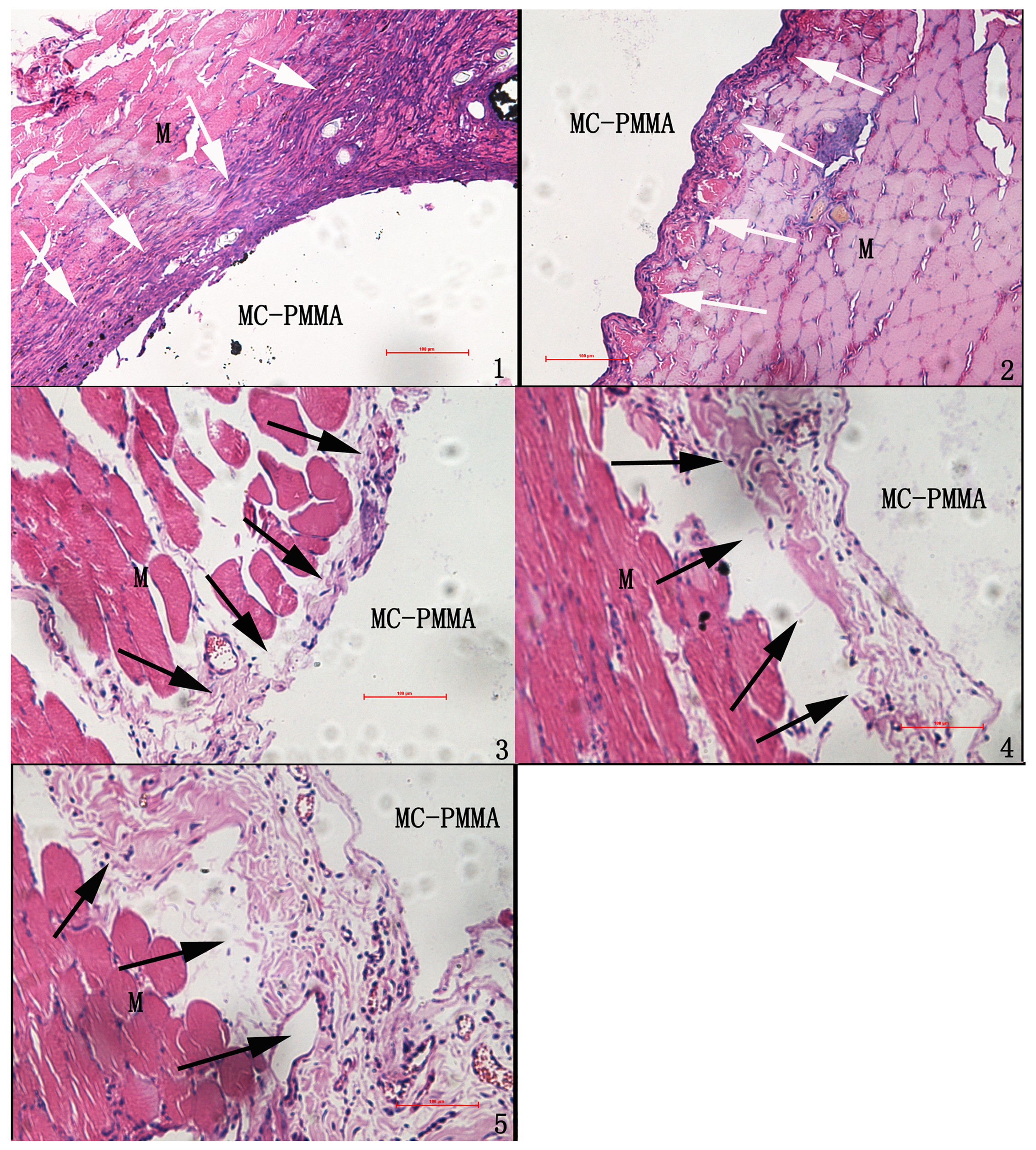

Supplement: S3 Fig — Bar = 100μm; M, muscle; white arrow, inflammatory cells; black arrow, connective tissue. (TIF) [file pone.0129018.s003.tif]

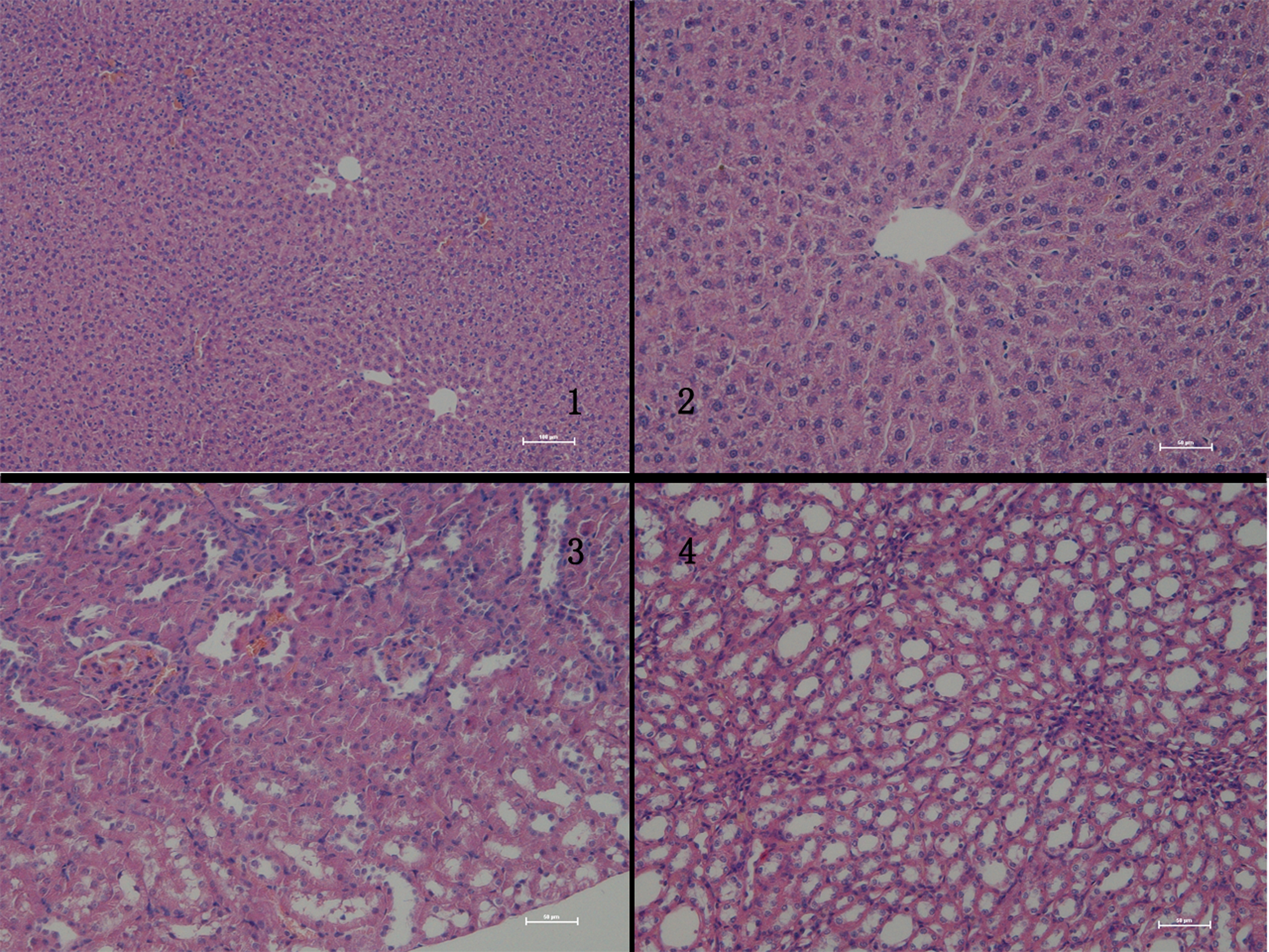

Supplement: S4 Fig — 1, 10×; 2–4, 20×. (TIF) [file pone.0129018.s004.tif]
