# Supplementary material for: Influence of Nano-HA Coated Bone Collagen to Acrylic (Polymethylmethacrylate) Bone Cement on Mechanical Properties and Bioactivity
Source: PLoS One. 2015 Jun 3;10(6):e0129018. doi: 10.1371/journal.pone.0129018 (PMC4454564; doi:10.1371/journal.pone.0129018)
Supplement: S1 Table — (DOCX) [file pone.0129018.s005.docx]

**Table S1. Information of gene primers**

| Gene | Length(bp) |  | Promer (5'-3') | Annealing temp. (℃) |
| --- | --- | --- | --- | --- |
| H-ACTIN | 317 | S: | CACCCAGCACAATGAAGATCAAGAT | 60 |
|  |  | A: | CCAGTTTTTAAATCCTGAGTCAAGC | 60 |
| H-SPARC | 136 | S: | GAAGCCCTGCCTGATGAGACA | 60 |
|  |  | A: | CCACCTCCTCTTCGGTTTCCTC | 60 |
| H-IBSP | 192 | S: | CTGCTACAACACTGGGCTATGG | 60 |
|  |  | A: | TTTCATCCACTTCTGCTTCGCT | 60 |
| H-COL1A1 | 136 | S: | GAAGACATCCCACCAATCACC | 60 |
|  |  | A: | TCTCGTCACAGATCACGTCATC | 60 |
| H-BGLAP | 263 | S: | TCACACTCCTCGCCCTATTG | 60 |
|  |  | A: | CTCCTGAAAGCCGATGTGGT | 60 |

S, sense primer; A, antisense primer.
