# Supplementary material for: Influence of Nano-HA Coated Bone Collagen to Acrylic (Polymethylmethacrylate) Bone Cement on Mechanical Properties and Bioactivity
Source: PLoS One. 2015 Jun 3;10(6):e0129018. doi: 10.1371/journal.pone.0129018 (PMC4454564; doi:10.1371/journal.pone.0129018)
Supplement: S2 Table — (DOCX) [file pone.0129018.s006.docx]

**Table S2**.The cell relative growth rate (RGR) with CKK8 method in different periods after cell subculture ($\bar{\boldsymbol{X}}$± SD (CTS), n = 6, %)

|  | 1^st^ day | 3^rd^ day | 5^th^ day | 7^th^ day |
| --- | --- | --- | --- | --- |
| PC | 3.31±0.18(4) | -0.15±0.14(5) | 0.36±0.62(4) | 0.38±0.70(4) |
| MC-PMMA | 93.99±4.37(1) | 104.35±7.82(0) | 114.52±6.60(0) | 100.00±10.29(0) |
| C-PMMA | 98.44±5.26(1) | 108.61±4.00(0) | 108.63±5.78(0) | 112.16±7.60(0) |

PC, Positive Control; NC: Negative Control; MC-PMMA: Mineralized Collagen PMMA Bone Cement; C-PMMA: Classical PMMA Bone Cement.
