# Supplementary material for: Influence of Nano-HA Coated Bone Collagen to Acrylic (Polymethylmethacrylate) Bone Cement on Mechanical Properties and Bioactivity
Source: PLoS One. 2015 Jun 3;10(6):e0129018. doi: 10.1371/journal.pone.0129018 (PMC4454564; doi:10.1371/journal.pone.0129018)
Supplement: S3 Table — (DOCX) [file pone.0129018.s007.docx]

**Table S3** The absorbance at 545nm wavelength (OD_545_) and hemolysis ratio ($\bar{\boldsymbol{X}}$± SD, n = 6)

|  | OD_545_ | HR（%） |
| --- | --- | --- |
| PC | 0.7999±0.0121 | 1 |
| NC | 0.0083±0.0004 | 0 |
| MC-PMMA | 0.0104±0.0006 | 0.26±0.08 |
| C-PMMA | 0.0097±0.0009 | 0.18±0.11 |

PC, positive control; NC, negative control; MC-PMMA: Mineralized Collagen PMMA Bone Cement; C-PMMA: Classical PMMA Bone Cement.
