# Supplementary material for: Influence of Nano-HA Coated Bone Collagen to Acrylic (Polymethylmethacrylate) Bone Cement on Mechanical Properties and Bioactivity
Source: PLoS One. 2015 Jun 3;10(6):e0129018. doi: 10.1371/journal.pone.0129018 (PMC4454564; doi:10.1371/journal.pone.0129018)
Supplement: S4 Table — (DOCX) [file pone.0129018.s008.docx]

**Table S4** Weight change after intraperitoneal injection of material extracts (gram, $\bar{X}$±SD, n=15）

|  | 24h | 48h | 72h |
| --- | --- | --- | --- |
| Control | 0.3327±0.2542 | 0.5527±0.4222 | 0.8220±0.4513 |
| MC-PMMA | 0.4387±0.3369 | 0.8133±0.6792 | 1.1473±0.9570 |
| p (t test) | 0.339 | 0.217 | 0.244 |
